# Medical and Surgical Society of Baltimore

**Published:** 1881-08

**Authors:** 


### MEDICAL AND SURGICAL SOCIETY OF BALTIMORE.

---

*Induration of Wrist and Palm.*—Dr. Taylor: This man, æt. 31, has an enlargement of the wrist, which I wish the gentlemen to examine. He is a cigarmaker, perfectly healthy, and there is no history of syphilis. These swellings have existed for more than a year. At first I was much puzzled, but now I think they are enlarged ganglia.

Dr. Chisolm: They come under the general head of ganglia. The contents of one can be pressed into the other, thus showing communication between the two. In a case of my own the contents were like grains of rice, and the swelling was tapped from time to time to exhibit the matter to the students. This case probably needs tapping and the injection of iodine for its cure.

*Reflex Paralysis.*—Dr. Caldwell: A gentleman, 40 years old, had partial paralysis of one side of the face; the corner of the mouth hung a little and there was some supraorbital pain, but most of the pain was in the perineum. A thorough examination was made and a portion of the prostate gland was found to be slightly enlarged and tender. I think the paralysis is dependent upon this irritation and enlargement, and is reflex in its character. He has been treated with galvanism, gelsemium, potass. brom. and tr. nux vomica, and is much improved. I rely upon galvanism to reduce the enlargement.

Dr. Arnold: A gentleman, over 50 years of age, had hemiplegia for two years and a half, and from concomitant symptoms I thought there was softening of the brain from embolus. There was retention of urine and when the catheter was used painful contractions of the right leg, with some oscillation and trembling, were produced, which would pass off when the catheter was removed. I attributed this to reflex action from irritation of the urethra. Hughlings Jackson, Nothnagel and Rosenthal are believers in reflex paralysis. That an irritation should produce an opposite effect, viz: paralysis, is the reason the efforts are made to explain by reference to other cases. It is difficult to understand how an enlargement of the prostate could produce facial paralysis. If it produced paralysis at all we would expect the lower limbs would be the parts to be affected.

Dr. Chisolm: In eye diseases we frequently have reflex symptoms; irritation of one eye producting paralysis in the other. By the removal of the diseased eye vision can be restored to the other in a few days.

*Sarcoma of Eyelid.*—Dr. Chisolm: A child, 5 years old, about one month ago was brought to the hospital with a tumor of the lid, which had been discovered only a few days before. The tumor was attached to the under surface of the lid by a pedicle containing large blood vessels. It was as large as a cherry, and full of blood vessels. No pain was produced by its presence. For fear the hemorrhage might be dangerous, I placed a ligature loosely around the mass in preference to removing it by the knife. On the next day it had not lost its color but was of a deeper red. Some of the blood vessels had given way and the bandage was soaked with blood. Nothing more was done at that visit beyond giving iron for the anæmic condition. In a few days the tumor was of a slightly ashy hue. Two days later it protruded between the lids, and a ligature was applied which cut through the mass and it fell off. The eye was examined and found to be in a healthy condition, except a slight ashy appearance of the cornea, for which lotion of boracic acid was used. Upon the child's return, two days later, the cornea was entirely eaten away. It is singular that the slight pressure of the tumor should produce changes. The tumor was examined microscopically and found to be a sarcoma containing both round and spindle cells, and is nearly certain to return. It is three weeks since the tumor was removed, and now it is nearly of the original size. The cornea has been replaced, but vision is lost and probably life may be destroyed. Billroth says these tumors are common except on mucous membrane, yet here we have a comparatively trivial growth threatening the life of the child.

*Abscess.*—Dr. Evans: Three or four months ago I was called to see a lady, aged 30; married; the mother of four children, one of whom, nine months old, was still at the breast; always had good health up to the present illness. After her confinements the menses usually appeared within six months, but at the time I saw her they had not been present since the birth of her last child. She was taken with a chill and severe abdominal pain, and a medical gentleman who attended her for eight weeks pronounced the case to be one of Bright's disease and said that it would be fatal. When I saw her there was great dyspnœa, anasarca and almost complete suppression of urine, half teacupful of coffee-colored urine being passed in the twenty-four hours. I feared to give her diuretics, but gave her one drop of croton oil every two hours until free watery evacuations were produced. In the evening she had passed a bucketful of water, and was much relieved. The next day I repeated the oil with manifest benefit; then ʒi. fl. ext. jaborandi, which produced profuse diuresis. The kidneys had recovered from their engorged condition and the urine flowed freely; then potass. bitart. was used to keep up the flow. All this time there was no pain, and the improvement continued for ten days, when the dropsy reappeared, and the croton oil was repeated for two days. She then complained of pain, and fluid ext. juniper was used as a diuretic. On the next morning she had a hemorrhage from the bowels and passed a large quantity of pus. The flow of pus continued for several days, but gradually lessened in quantity. I think she passed several gallons. She made a good recovery and is now well. I do not think she had Bright's disease.

Dr. Percivall: I do not think it was abscess of the kidney. The wife of a great friend of mine, two weeks after her second confinement, discharged a quart of most offensive pus, which, I think, came from the fallopian tube. She got well, but had no more children. I had a negro woman who died, and upon post mortem an abscess of the fallopian tube was found which contained two quarts of pus. These abscesses are sometimes two or three months in forming.

Dr. Rohe: It is a pity that the early history of Dr. Evans' case is not clearer. As it occurred subsequent to partruition it seems likely that it was a pelvic abscess. It is not unusual for these abscesses to occur in this location, and several distinguished gynæcologists have diagnosed ovarian tumors which afterwards proved to be abscesses.

Dr. Arnold: It is curious that the first physician diagnosed Bright's disease, and that Dr. Evans did not make a diagnosis is equally strange. Abscesses following partruition are very common, and the collection of pus might press on the abdominal vessels and produce anasarca. I recall a case which I saw in consultation with Dr. Regester, in which there were rigors, sweats, anasarca in the lower extremities and some albumen in the urine. The abscess broke and large quantities of pus were discharged. The venous congestion produced the albuminuria.

Dr. Evans: The woman was perfectly well for nearly nine months after her delivery. It would be rather singular for an abscess to exist that length of time and show no signs of its existence.

Dr. Rohe: There are cases on record which lasted one or more years without constitutional symptoms pointing to collection of pus.

Dr. Chambers: If it was a pelvic abscess we might expect to find dropsy of the lower extremities, but it does not explain why the upper extremities were also dropsical.

Dr. Reid: It is fortunate that Dr. Evans did not make a diagnosis. If he had been sure that it was a pelvic abscess, he might have called in a gynæcologist to aspirate or a surgeon to open with his bistoury, and the result might not have been so favorable.

*Opium Poisoning.*—Dr. Chambers: I was called to see a lady who had taken ʒ i. of laudanum; gave atropia gr. half, and she got well. There was but little walking and slapping and therefore not much exhaustion. I gave a man two grains of atropia in eight hours and he recovered.

Dr. Taylor: In the case of the lady the druggist suspected her of intending suicide and gave plaster of Paris instead of arsenic, and the laudanum was very much diluted.

Dr. Chambers: She had the physiological symptoms of opium, and it is not important whether she had taken it or not. There was in point pupil. pulse 50, respiration 8. The atropia was given to produce its physiological effects.

*Incipient Inguinal Hernia.*—Dr. Monmonier: I was called in consultation by Dr. Brinton to see a man seventy-five years old, who was suffering from obstruction of the bowels, vomiting of fecal matter, etc. Upon examination no swelling was found at the external abdominal ring; some slight œdema; no globular mass could be detected; no tympanitis. The finger could be passed through the external ring into the inguinal canal. The diagnosis of strangulation at the internal ring was made and an operation advised. The doctor did not think an operation was desirable then, and the mattter was postponed for a few hours, during which the man died. Upon post mortem examination the small intestine was found fixed in the internal ring. The bowel was much contracted, a condition frequently seen in the aged. The constriction did not embrace the whole calibre of the bowel, about one-third of the circumference was firmly pinched by the ring. There was no peritonitis; the man died from exhaustion.

Dr. Brinton: The reasons why I did not acquiesce in Dr. Monmonier's desire to operate were that the patient was very much opposed to any such measures, and the doctor and myself were not positive that it was strangulated hernia. I thought a few hours' delay would be better. The diagnosis was not sure until late in the evening, and then the patient was very much depressed and died shortly afterwards.

*Abscess of Tonsil.*—Dr. J. H. Hartman: A young adult had severe pain in deglutition and a burning feeling in his throat. His father thought he had quinsy, and underthe use of gargles and domestic remedies he seemed to be doing fairly until a day or two before he came to me. The left tonsil was swollen as large as a pigeon's egg and there appeared to be deep seated inflammation. The swelling was opened freely and much pus escaped. Further inspection revealed a white substance in the tonsil which, upon extraction, proved to be a piece of wooden toothpick about a half-inch long. This is the first case of the kind that has come under my observation, although many cases are reported in the books. Fish bones, toothbrush bristles, apple seeds, etc., have been found in the tonsils and have produced abscess. I have frequently, in excising the tonsil, met calcareous concretions, sometimes as large as a bean. More than once I have been compelled to remove the tonsilotome and finish the operation with a bistoury.

Dr. Cathell: Under what circumstances does Dr. Hartman decide to remove the tonsils?

Dr. Hartman: I never remove the acutely conjested tonsil. In chronic hypertrophy, if the tonsil project half an inch beyond the arches of the palate, it should be removed. The operation is not troublesome, and the hemorrhage is slight. I have operated nearly three hundred times.

*Neuralgia.*—Dr. Brinton: I attended a lady in her second confinement. The labor was natural and not severe. The day after her delivery an intense neuralgia of the fifth pair of nerves appeared and has continued in spite of all treatment. The pain appears at 8 o'clock every morning and lasts until 3 in the afternoon. I gave quinine in ten-grain doses four times a day without relief. Aconite, belladonna, chloral and gelsemium have been used. I gave ʒ i. of tr. gelsemium every two hours without effect. Thinking the preparation inert, Squibb's fl. ext. was used in five-drop doses, gradually increased to twenty drops. Of this ʒ iiss. were used before the physiological effects were observed.

Dr. Morris: In a similar case, in which a lady had an attack every morning, I used quinine in small doses without effect, but giving twenty grains one hour before the expected seizure promptly cured it.

Dr. Hartman: Ringer recommends tonka. I have not used it.

Dr. Brinton: I gave quinine in ten-grain doses, frequently repeated; had cinchonism, but no cure of the neuralgia.

Dr. Morris: There may be some inflammation or interference with the nerve. In a case in which everything had been tried relief was obtained from potass. iod. and bichloride of mercury.

Dr. Ashby: If dependent upon syphilis, potass. iod. will relieve. Tonka is a remedy largely used by the Feejees; it is a combination of leaves. It is highly recommended, but personally I know nothing of its value.

Dr. Monmonier: Beard recommends phosphate of zinc. I have used salycilate of cinchonidia with benefit.

Dr. Chambers: As an empirical remedy I think Fowler's solution hypodermically has much to recommend it. The periodicity in this case is against the opinion that there is inflammation; there would also be some tenderness.

Dr. Scarff: In one of my cases facial neuralgia came on just before the child was born and lasted four days. It was relieved by ergot and hyoscyamus.—*Maryland Medical Journal.*

